# Cholesterol modulates vesicle clustering mediated by alpha-synuclein in a nonlinear fashion

**DOI:** 10.52601/bpr.2024.240047

**Published:** 2025-12-31

**Authors:** Owen Tyoe, Chinta Aryal, Jiajie Diao

**Affiliations:** 1 Department of Physics, University of Cincinnati College of Arts and Sciences, Cincinnati, OH 45221, USA; 2 Department of Cancer and Cell Biology, University of Cincinnati College of Medicine, Cincinnati, OH 45267, USA

**Keywords:** Proteins, Lipids, Membranes, Cholesterol, Vesicles

## Abstract

α-Synuclein (α-Syn) is a presynaptic protein primarily associated with Parkinson’s disease and other neurodegenerative diseases. The cholesterol content in SV membranes regulates α-Syn binding to synaptic vesicles, changing its function and modifying its aggregation. Using single-vesicle imaging, we show that low concentrations of cholesterol reduce vesicle clustering, and high concentrations enhance vesicle clustering mediated by α-Syn. Furthermore, using all-atom molecular dynamics simulation, we investigate the role of cholesterol in synaptic-like vesicle clustering mediated by α-Syn. In particular, we found cholesterol reduces hydrogen bonds and interaction energies in low concentrations, while high concentrations of cholesterol increase hydrogen bonds and interaction energies. Moreover, cholesterol also regulates lipid packing defects, and the condensation of cholesterol leads to the suppression of shallow packing defects, and enhancement of large defects with increasing cholesterol concentration. We revealed that cholesterol promoted vesicle clustering is due to the electrostatic interaction between cholesterol in the membrane and the N-terminal region of α-Syn. Moreover, this increased electrostatic interaction arises from a change in packing defect distribution of the protein–membrane interface induced by cholesterol condensation. This work highlights the complex interplay between α-Syn and cholesterol, emphasizing the importance of cholesterol levels in membranes and their impact on α-Syn function.

## INTRODUCTION

α-Syn is a small, soluble protein consisting of 140 amino acids, which is mainly expressed in neuronal cells and predominantly localizes at presynaptic termini (Maroteaux *et al*. 1988). α-Syn has gained significant attention in the field of neuroscience due to its association with several neurodegenerative disorders, most notably Parkinson's disease (PD). In pathological conditions including PD, α-Syn misfolding can lead to membrane disruption and its aggregation into small, toxic oligomers (Conway *et al*. 2000; Spillantini and Goedert 2000). These oligomers are believed to be more harmful to neurons than the monomeric form and may disrupt normal cellular functions; in particular, oligomeric or fibrillar α-syn is associated with more rapid destruction of the membrane than soluble monomeric α-synuclein (Zhu *et al*. 2003). These insoluble fibrils are a major component of Lewy bodies and are associated with cell toxicity and neurodegeneration in conditions like PD (Necula *et al*. 2003; Serpell *et al*. 2000; Zhu *et al*. 2003). Enhanced aggregation and fibrilization and corresponding pathology is associated with mutations including A30P and A53T (Jensen *et al*. 1998; Kruger *et al*. 1998), but further familial forms of PD are associated with the point mutations E46K, and H50Q, while artificial point mutations E35K and E57K also increase oligomerization (Tsigelny *et al*. 2015). These aggregates are a hallmark feature of the disease and are believed to contribute to the death of dopaminergic neurons in the brain, leading to the motor symptoms of Parkinson's disease, such as tremors, rigidity, and bradykinesia (slowness of movement) (Goedert 2001).

The physiological function of α-Syn is still under investigation, but it is thought to play a role in synaptic plasticity, regulating synaptic vesicle trafficking and neurotransmitter release, and exocytosis and endocytosis (Clayton and George 1998, 1999; Diao *et al*. 2013; Lautenschlager *et al*. 2017; Liu *et al*. 2004; Wang *et al*. 2016). α-Syn is comprised of three distinct regions: the N-terminal region, the central hydrophobic NAC region, and the C-terminal acidic region (Clayton *et al*. 1998; Wang *et al*. 2016). The amphipathic lysine-rich N-terminal domain is involved in anionic membrane interactions and adopts a highly helical conformation which is associated with binding to lipid vesicles; the non-amyloid beta component (NAC) which is also associated with SV interaction and clustering, this region is associated with the formation of pathological aggregates; and a C-terminal domain associated with VAMP2 interactions (Clayton *et al*. 1998; Diao *et al*. 2013; Eliezer *et al*. 2001). α-Syn is involved in synaptic function by assisting in SNARE-complex assembly and SV clustering (Diao *et al*. 2013). The clustering of synaptic vesicles (SVs) and promotion of SNARE formation, relies on its interaction with phospholipid membranes. The N-terminal domain of α-Syn is well known for having a helical or broken-helical structure whose residue sequence is associated with membrane curvature and packing defect sensing (Lokappa and Ulmer 2011; Jensen *et al*. 2011; Middleton and Rhoades 2010; Nuscher *et al*. 2004). In particular, strong membrane binding is driven by electrostatic interaction between the negatively charged bilayer and positively charged N-terminus of α-Syn, and its affinity for highly curved lipid surfaces, which may be modulated by lipid composition and the presence of bilayer defects (Middleton and Rhoades 2010).

In a previous study, using a solid-state nanopore system we found that N-terminal acetylation significantly decreases α-Syn oligomerization; furthermore, replica-exchange molecular dynamics simulations revealed that the addition of an acetyl group at the N-terminus disrupts intermolecular hydrogen bonds, which slows down oligomerization (Bu *et al*. 2017). Additionally, using all-atom molecular dynamics simulations, we found that O-GlcNAc modifications can suppress the oligomerization of α-Syn aggregates through a steric effect, in particular, the O-linked glycosyl group disrupts the formation of hydrogen bonds between α-Syn monomers (Wu *et al*. 2020). Beyond Parkinson's disease, α-Syn aggregation has been implicated in other neurodegenerative diseases, including Dementia with Lewy Bodies (DLB) and Multiple System Atrophy (MSA) (Spillantini and Goedert 2000).

Variations of cholesterol levels are involved in the pathogenesis of neurodegenerative diseases including Alzheimer’s and Huntington's disease (Cutler *et al*. 2004; Valenza *et al*. 2007). It has been shown *in vivo* that MβCD-induced cholesterol depletion reduces basal synaptic transmission in the CA1 area in a time-dependent manner (Frank *et al*. 2008). Several studies carried out in synaptosomes and PC12 cells suggest that lipid rafts are highly enriched with SNARE proteins (Chamberlain *et al*. 2001; Lang 2007) and that cholesterol depletion greatly reduces calcium evoked neurotransmitter release from synaptosomes (Chamberlain *et al*. 2001; Gil *et al*. 2005). Using exchange and transverse relaxation NMR and CD spectroscopy, DLS and cryo-EM, recent studies have shown that cholesterol enhances vesicle-vesicle interactions mediated by α-Syn, and furthermore, cholesterol mediates binding affinity of the NAC region of α-Syn (Man *et al*. 2020).

Another recent study showed that α-Syn amyloid oligomers can cause hemifusion in negatively charged vesicles (Stefanovic *et al*. 2015). Furthermore, Fantini *et al*. (2013) show the NAC region (of α-Syn) has a high affinity for cholesterol, and induces folding upon interaction, and the tilted geometry of the cholesterol/α-Syn complex facilitates the formation of oligomeric (ion) channels (Fantini and Yahi 2013). Moreover, in the presence of cholesterol, α-Syn oligomers induce membrane disruption, via increased rigidity and dehydration of the membrane (van Maarschalkerweerd *et al*. 2015). Jakubec et al showed cholesterol inhibits α-Syn interaction with lipid bilayers (dependent on lipid composition), and promotes α-Syn fibrillation, using lipid nanodiscs. Particularly for DOPC only nanodiscs, additional cholesterol modulates the interaction of the NAC region, so that the NAC region has promoted interaction with the bilayer. While for DOPC + DOPE + DOPG nanodiscs, the NAC region is unaffected, but N- and C-termini binding is inhibited (for nanodiscs containing 30% cholesterol compared to 0%) (Jakubec *et al*. 2021).

Leftin *et al*. used Solid-state ¹³C NMR to show that α-Syn induces thinning in the membrane, and increases lipid cross-sectional area (Leftin *et al*. 2013). This perturbation implies structural membrane remodeling of a raft-like liquid-ordered phase. Previous work also showed cholesterol suppresses membrane leakage by decreasing water penetrability, so that increasing cholesterol reduces leaky membrane fusion (Bu *et al*. 2018). Moreover, α-Syn has increased interaction with smaller, highly curved lipid membranes containing more packing defects, and the number of MB packing defects is proportional to curvature and cholesterol, and defect size is also regulated by cholesterol (Liu *et al*. 2021).

Cholesterol has many interactions and is abundant in many physiological processes, but it is not as clear the role of variable cholesterol in specific protein–membrane interactions and the transition from physiological to pathological biophysical interactions. In particular, while some aspects of cholesterol interacting with binding proteins and lipid membrane structures have been discussed above, there are no present studies quantifying docking of alpha-synuclein on synaptic-like vesicles and alpha-synuclein-induced vesicle clustering, with respect to varying concentrations of cholesterol. We show that increasing concentrations of cholesterol have a nonlinear influence on energy associated with binding as well as protein-induced vesicle clustering. Specifically, we show high concentrations of cholesterol enhance membrane binding and protein-induced vesicle clustering, while low concentrations have an inhibitory effect. We attribute this effect to the effect of cholesterol condensation which increases with concentration, rather than pure lipid/sterol diffusion. Cholesterol modulates the curvature and distribution of packing defects in lipid membranes, which further be perturbed upon protein interaction, and this behavior is nonlinear. The fact that this implicates steps in the process of neurotransmission, and that lipid expression is affected by neurodegenerative diseases suggests the effect of cholesterol on physical membrane properties is highly dynamic and can regulate diffusion of membrane components, and given its role in membrane fluidity, rigidity, and permeability, as well as interactions with (monomeric or multimeric) proteins and other structures associated with vesicle docking, clustering, and fusion, our work suggests (interactions of) cholesterol may be implicated in several physiological or pathological processes in multiple neurodegenerative diseases.

## RESULTS

### Vesicle clustering mediated by α-syn is modulated by cholesterol

We follow a similar protocol as used in previous studies (Aryal *et al*. 2021; Tyoe *et al*. 2023). Here we report the role of cholesterol in the interaction of SV-like vesicles and α-Syn, where SV-like vesicles are composed of phospholipids including DOPC, DOPE and DOPS. For this we considered three variations in concentration of cholesterol, fixing 12% PS in all cases (see Table 1), and imaging was performed via TIRF microscopy. We use 12% PS vesicles (0% mole cholesterol) as a control group, whereas 10% and 40% mole are the representative small and large concentrations of cholesterol respectively.

**Table 1:** Lipid composition (%mole) for a given type of vesicle

Lipids/Dyes	DiD vesicles	DiI 0% CHOL	DiI 10% CHOL	DiI 40% CHOL
DOPC	66.8	67	57	27
DOPE	20	20	20	20
DOPS	12	12	12	12
CHOL	0	0	10	40
Biotin-PE	0.2	0	0	0
DiD	1	0	0	0
DiI	0	1	1	1
Note: DiD labelled vesicles and DiI labelled vesicles are read as DiD-vesicles and DiI-vesicles				

**Figure 1:**
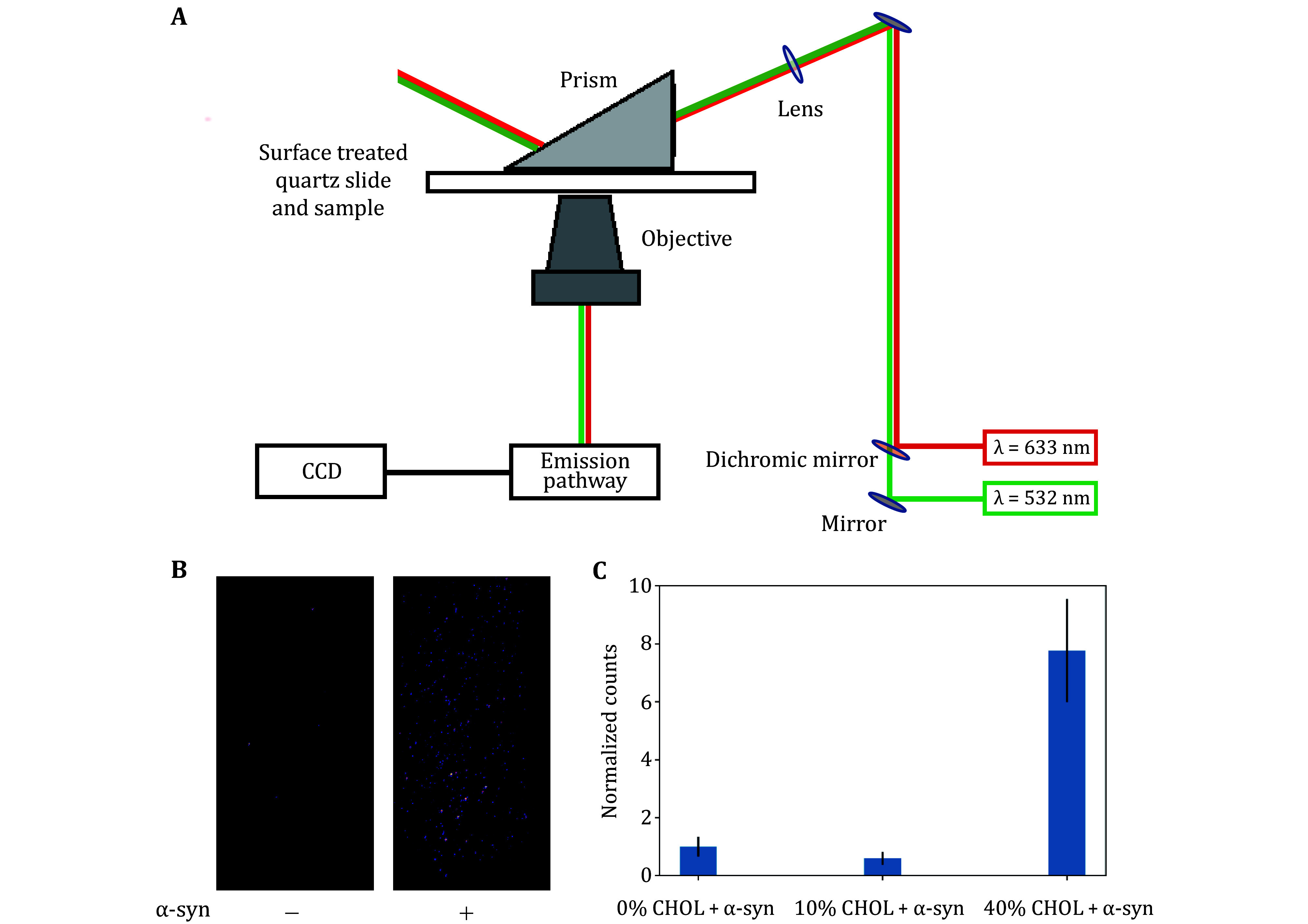
Schematic of TIRF microscope set-up for vesicle clustering measurements. **A**,**B** Vesicle clustering caused by α-Syn (**A**) and representative TIRF image of vesicle clustering (**B**) of the following vesicle compositions: 12% DOPS + 20% DOPE + 48% DOPC + 0% CHOL (left) in the absence and (right) in the presence of 250 nmol/L α-Syn. **C** The enhancement in vesicle clustering caused by 250 nmol/L α-Syn in 12 mol% PS + variable mol% CHOL vesicles

Figure 1A shows the experimental setup for TIRF imaging. Figure 1B shows the representative TIRF images of the effect in vesicle clustering caused by α-syn without cholesterol, and Fig. 1C shows the resulting clustering data in all three cases of cholesterol concentration. From clustering data given by 12% PS + 0% cholesterol DiI vesicles, we find vesicle clustering is enhanced via α-syn binding as expected. The vesicle clustering count of 10% cholesterol is slightly decreased, and significantly increased in the case of 40% cholesterol in the presence of α-syn, as compared to 0% cholesterol. Control groups (12% PS + 0% cholesterol) were considered when the change caused by α-Syn was significant (*p* < 0.001%) as compared to the background, without α-Syn.

**Figure 2:**
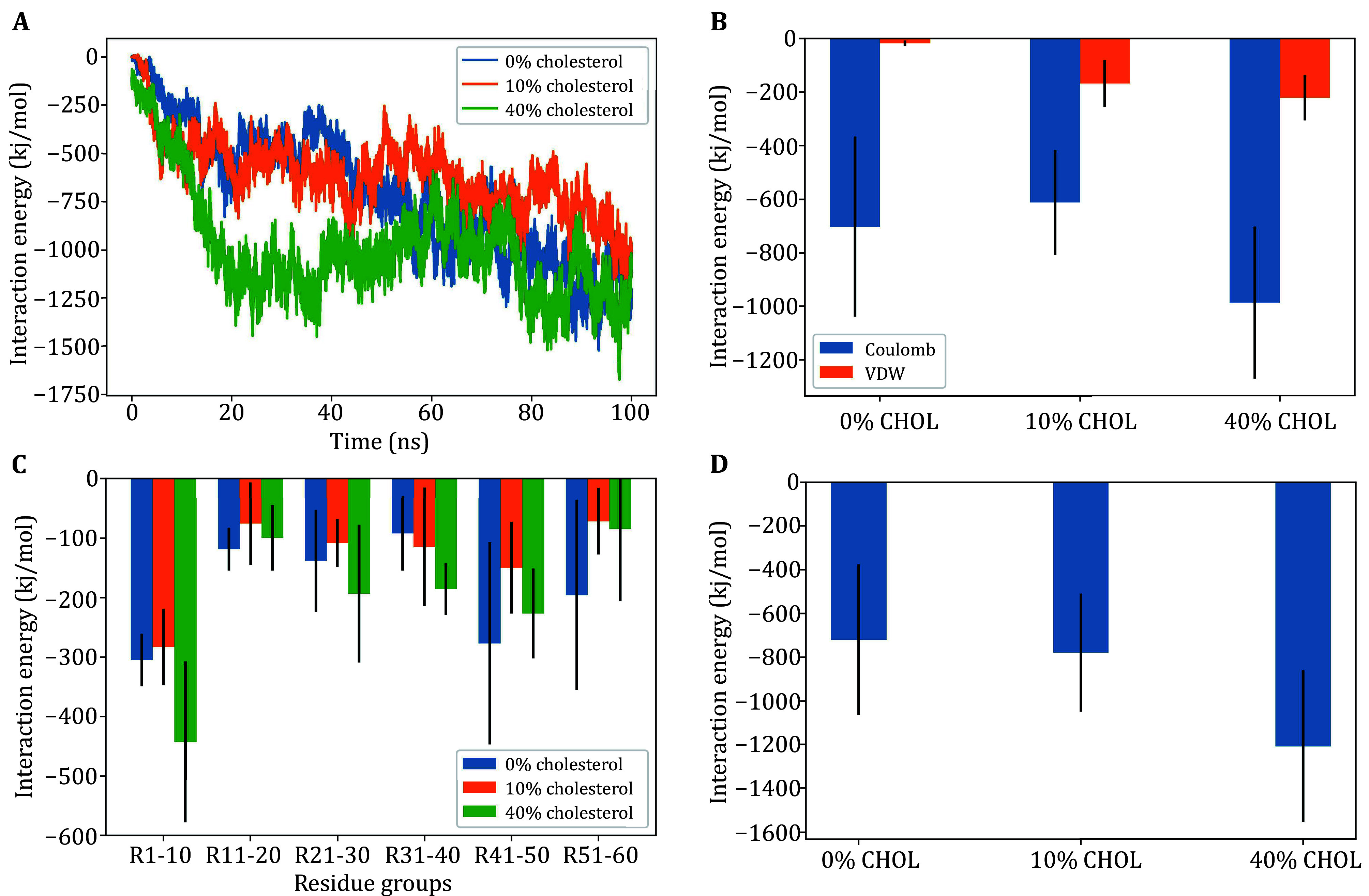
**A** Ensemble average of combined electrostatic and hydrophobic interaction energy as a function of time, with and without cholesterol. **B** The average interaction energy per lipid, split into the electrostatic (Coulomb) and hydrophobic (van der Waals) interactions. **C** The average Coulomb interaction energy per residue group, split into residues 1–10, 11–20, 21–30,31–40, 41–50, 51–60. **D** The average energy corresponds to the ensemble average of combined interaction energy, for each system

For all vesicle compositions, 12% PS was maintained. Furthermore, for the enhancements in clustering caused by α-Syn for 10% cholesterol and 40% cholesterol (compared to 0% cholesterol), we have *p* < 0.01% and *p* < 0.01% as shown in Fig. 2C. Thus, our results reveal a nonlinear relationship in the role of concentration dependence of cholesterol in vesicle clustering; that is, cholesterol enhances vesicle clustering mediated by α-Syn in large concentrations, but inhibits vesicle clustering (mediated by α-Syn) at low concentrations.

### Cholesterol modulates electrostatic α-Syn–membrane interaction

We performed molecular dynamics (MD) simulations to study the interaction of α-Syn and lipid membranes with and without cholesterol. Other studies have used MD simulations to study the structural properties of α-Syn upon membrane binding, the role of calcium ion influx and charge screening effects, and the influence of membrane curvature and lipid packing defects on α-Syn binding (Cai *et al*. 2019, 2020; Jensen *et al*. 2011; Liu *et al*. 2021; Perlmutter *et al*. 2009; Vermaas and Tajkhorshid 2014).

**Figure 3:**
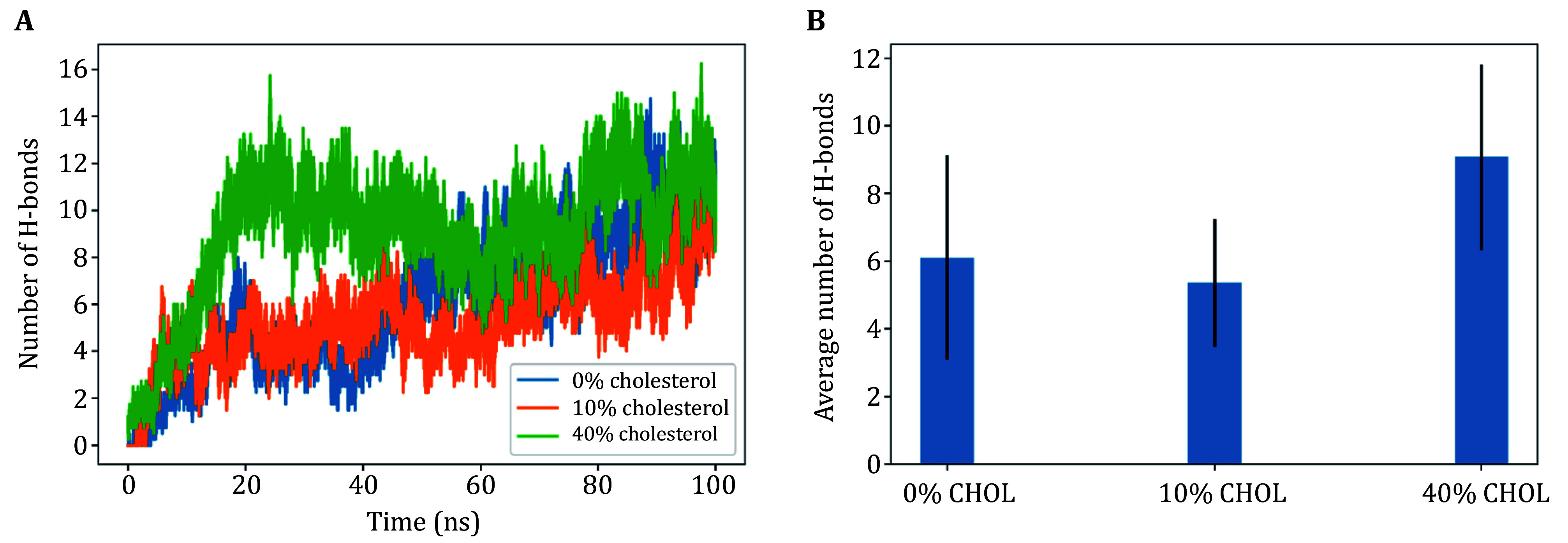
**A** Total number of hydrogen bonds between α-Syn and the lipid membrane as a function of time (averaged over four runs each), with and without cholesterol. **B** Average number of hydrogen bonds between α-Syn and membrane, with and without cholesterol

Using all-atom simulations, we can study the embedding of α-Syn’s N-terminal region given by amino acids 1–60, to a model anionic membrane of similar SV-like composition with variable cholesterol. Figures 2–3 show the total interaction energy and total number of hydrogen bonds between α-syn’s N-terminus and lipid membranes. Figures 2A–2D illustrate the details of the interaction energy between α-syn’s N-terminus and lipid membranes of varying composition. Figure 2A illustrates the α-syn–membrane (ensemble average) interaction energy as it varies over the simulation runtime. Figure 2B shows the contributions of electrostatic interaction energy and hydrophobic interaction energy in the protein–membrane system, and confirms that electrostatic interaction is the primary driving force of their binding. Figure 2C shows how the average interaction energy is partitioned among the first 60 residues, in particular, the total protein–membrane interaction energy of 10 residue sections of α-syn. Consistent with the results of the clustering experiments (Fig. 1), the MD results showed that high concentrations of cholesterol enhance α-syn binding to (anionic lipid) membranes, while low concentrations have an inhibitory effect, as seen in Figs. 2D and 3B. Consistent with the clustering experiments (Fig. 1C), the MD results showed that high concentrations of cholesterol enhance α-Syn binding to (anionic lipid) membranes, while low concentrations inhibit vesicle clustering. We discuss more on the mechanism in the next section.

### Number and size distribution of membrane packing defects are regulated by cholesterol

**Figure 4:**
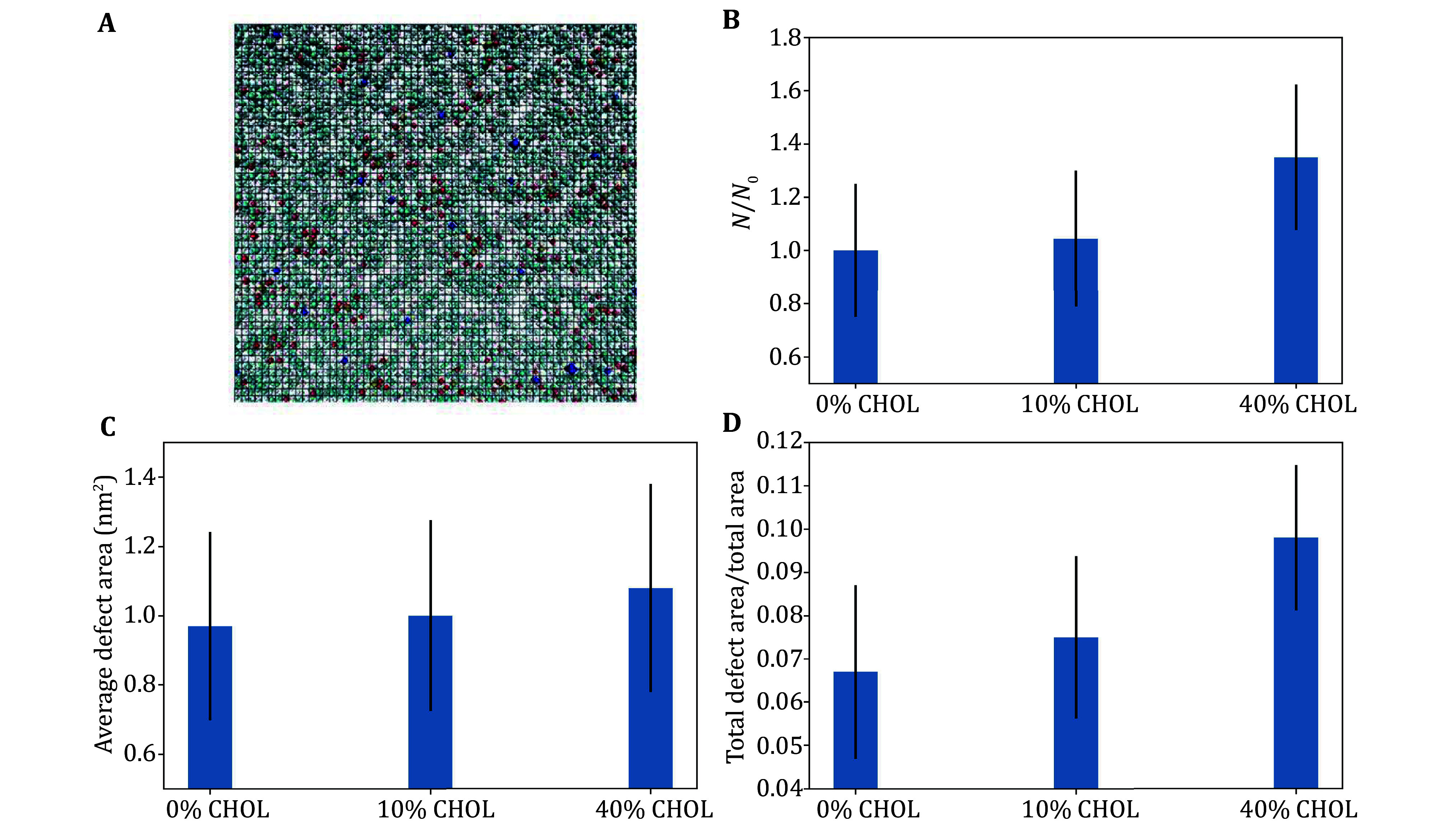
**A** Graphical representation of the calculation of membrane packing defect. **B** The ratio of the total number of membrane packing defects relative to the total for a lipid membrane without cholesterol. **C**,**D** The calculations for average area (**C**) and total area (**D**) of defects

We quantify the effect on the defect area fraction and number distribution, similarly as reported in a previous study (Liu *et al*. 2021). Our previous results suggest cholesterol regulates membrane curvature via packing defects, in particular, the number of membrane defects is proportional to the curvature and the size can be regulated by cholesterol. Furthermore, the nature and initial abundance of packing defects, curvature, and other properties which modulate the binding of α-Syn to model membranes are dependent on the lipid composition (de Jesus *et al*. 2013; van den Brink-van der Laan *et al*. 2004; Vamparys *et al*. 2013). That is, depending on lipid composition, larger defect number distributions and area fractions correspond to enhanced protein-membrane binding, via enhanced hydrogen bonds and binding energy (Liu *et al*. 2021). Figure 4 shows this is indeed the case for our model membrane systems with the N-terminal region of α-Syn.

The mechanism can be understood as follows. For large concentrations, cholesterol enhances the protein binding through increasing the hydrogen bond interactions between the protein and lipids. The protein firstly diffuses to the membrane surface anchored by the formation of hydrogen bonds available from cholesterol condensation associated with a packing defect. Since the protein is more likely to diffuse to a packing defect region, the cholesterol molecules are more likely to form hydrogen bonds directly with α-Syn, thereby increasing electrostatic interaction energy. Hence, cholesterol enhances the protein binding by increasing the hydrogen bond interactions between the proteins and cholesterol at high concentrations of cholesterol. However, this does not explain the inhibited interaction for the case of 10% cholesterol, furthermore, this definition of packing defect is relative and is dependent on a choice of threshold, for us the threshold is given by the angle between two lipid headgroups relative to one of the lipid acyl chains, as represented on a mesh grid which also depends on a choice of grid size. We resolve this issue by calculating the packing defect size constant, which depends on the distribution of defects as a function of defect size (threshold), and so is independent of this choice.

**Figure 5:**
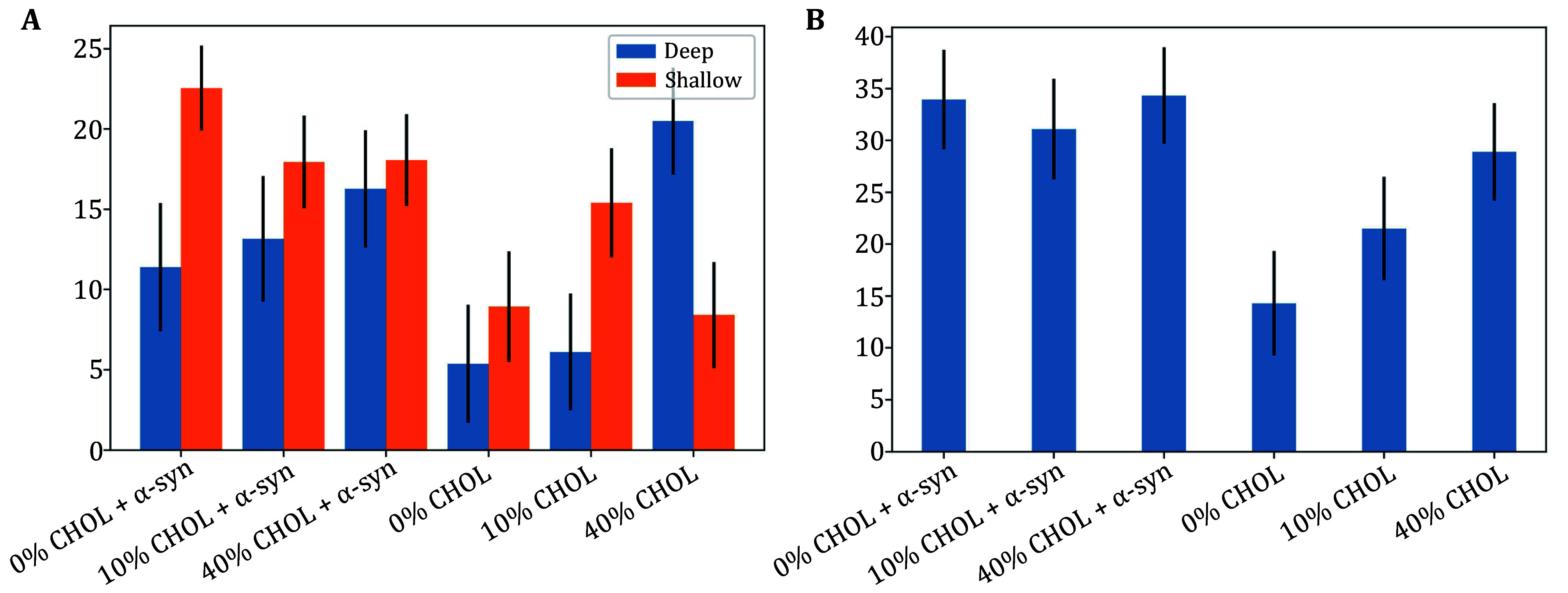
**A** Defect size constant, comparison for deep versus shallow packing defects. **B** Defect size constant, total, deep and shallow defect size constant. Here 0% CHOL + α-Syn means 0% Cholesterol and α-Syn, and 0% CHOL means 0% cholesterol, membrane only (without α-Syn)

**Figure 6:**
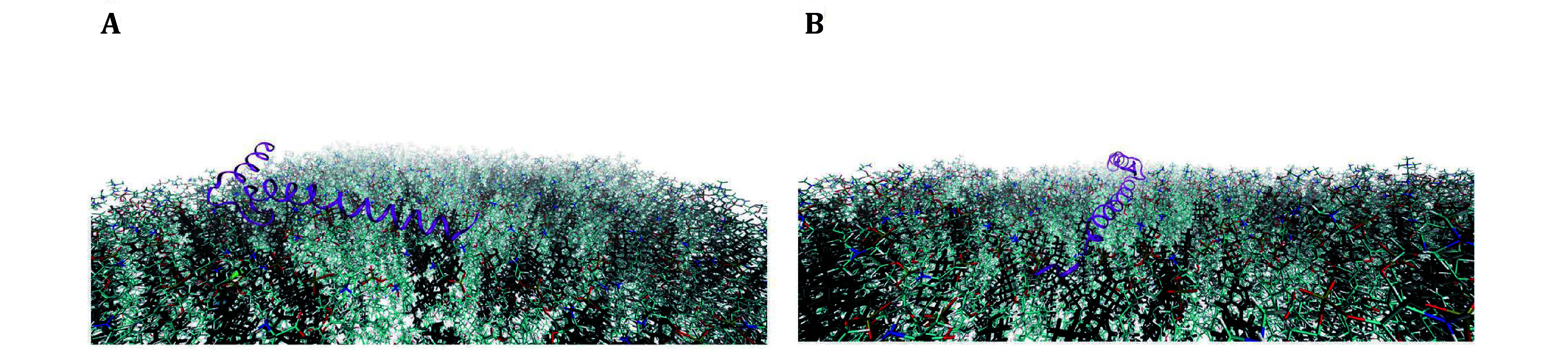
Snapshots of α-synuclein-cholesterol contacts, during the MD simulation. Panel A shows strong protein–membrane interaction via residues 1–10 and 41–50 (**A**), while Panel B shows the embedding of the N-terminal region and interactions of residues 1–10 with cholesterol in membrane (**B**). In the membrane, cyan represents C–C bonds, red represents O-bonds, orange P-bonds, blue N-bonds, white H-bonds, and cholesterol is represented in black. This particular membrane is composed of 12% DOPS, 20% DOPE, 40% CHOL and 28% DOPC, corresponding to 40% cholesterol

We next discuss the method to determine defect size constant, to further quantify and distinguish between shallow and deep membrane packing defects. A depth of 1 Å below the glycerol region is used to define the threshold between defects, *i*.*e*. deep vs shallow (Ulmer *et al*. 2005). The packing defect constant, *A*_0_, in units of Å^2^, is used as a parameter to quantify the interfacial packing defects in lipid bilayers (Manna and Murarka 2021). Even though our definition and calculation of packing defects is different from PackMem, our results are consistent with the results from Qi *et al*. (2023). In particular, Fig. 5 shows increasing concentrations of cholesterol enhances deep packing defects, but suppresses shallow packing defects, consistent with condensation. This means at low concentrations of cholesterol, the suppression of shallow defects due to cholesterol condensation means the protein is less likely to diffuse to a (deep) membrane packing defect, and hence we see a reduced interaction. We also note that the results shown in Fig. 5 with regard to deep packing defects are consistent with the results shown in Fig. 4. Figure 6 shows protein–cholesterol contacts during the MD simulation, where cholesterol condensates enhance hydrogen bonding with α-Syn; here cholesterol is represented in black, and α-Syn in purple. The results suggest a more significant effect of cholesterol on the interaction of the protein with the membrane, *i*.*e*., α-Syn interaction affects the distribution of lipids in the membrane, but membrane properties including lipid packing, diffusion, and rigidity are modulated by cholesterol and determine membrane rigidity, charge and other properties) affect the binding and conformational properties of α-Syn.

## DISCUSSION

A single-vesicle clustering assay revealed that vesicle clustering with α-Syn varies by concentration of cholesterol in a nonlinear fashion. Cholesterol enhances vesicle clustering with α-Syn for high concentrations, and inhibits vesicle clustering for low cholesterol concentrations, which can be attributed to the change in the membrane microenvironment caused by cholesterol. In particular, high concentrations of cholesterol lead to increased cholesterol condensation which changes the membrane packing defect distribution, such that α-Syn can embed more quickly, and thus more effectively mediate vesicle clustering. This result is consistent with the result of Qi *et al*. (2023), who showed cholesterol assists the membrane insertion of α-Syn by modulating the macroscopic membrane properties and defect distribution.

Furthermore, MD simulations also indicated that cholesterol enhances vesicle association of α-Syn at high concentrations via enhanced electrostatic interaction energy, and inhibits electrostatic interaction at low cholesterol concentrations. Explicitly, we find cholesterol significantly enlarged defect area fraction, by increasing the total number of defects and average area of one defect, with these both increasing proportionally to cholesterol. However, the defect area constant suggests that while deep defects are enhanced, shallow defects are suppressed for increasing concentrations of cholesterol. This suggests the lateral diffusion and condensation of cholesterol may lead to a connection of smaller defects into larger defect regions. The larger defect area would expose more neighboring acyl tails, which may enhance hydrophobic interaction which allows for increased binding, and additionally, interaction with -OH headgroups (from increased condensation of cholesterol) leads to increased hydrogen bonding with α-Syn.

However, we note that Mahapatra *et al*. showed the opposite trend, in particular, their results suggest that the membrane binding affinity of α-Syn is lower for 0 and 40% cholesterol, and maximal at 10% cholesterol (Mahapatra *et al*. 2021). We contribute this effect to the difference in protein-to-lipid ratio. For our clustering experiments, we used protein-to-lipid ratios corresponding to physiological α-Syn-SV binding obtained from the experiment (Fakhree *et al*. 2016).

Our results suggest that the mechanism of cholesterol in regulating vesicle clustering varies with the cholesterol concentration, but we do not claim to have the optimal value for cholesterol concentration leading to a minimum binding affinity and furthermore, we do not know if it is unique. However, our results are consistent with other experiments and studies using MD simulation (Liu *et al*. 2021; Qi *et al*. 2023), and these suggest that low (nonzero) cholesterol concentrations reduce membrane binding affinity of α-Syn (and possibly increase membrane stability to perturbation), and this concentration-dependent effect is what leads to increased or decreased vesicle clustering mediated by α-Syn, although more research is needed to determine the nature of this relationship, and what concentration (possibly) gives minimal vesicle clustering. This work supports other results suggesting that even though α-Syn membrane binding is electrostatically driven, neutral molecules can have a significant effect on the interaction and conformation of α-Syn (Lai *et al*. 2023; Tyoe *et al*. 2023).

Our results, along with other recent studies reveal the dynamics of membrane packing defects and electrostatic effects in regulating the folding of the N-terminus of α-syn and its interaction with membranes (Liu *et al*. 2021; Tang *et al*. 2024). It is also consistent with results from McClain *et al*., which suggest that binding affinity is enhanced with increasing cholesterol (McClain *et al*. 2023). Kang *et al*. used all-atom MD simulations to study synaptic vesicles and the effect of curvature on the membrane dynamics and lipid nanodomain organization, and their results suggest that curvature may induce phase separation in an otherwise fluid, disordered membrane (Kang *et al*. 2024). Moreover, our study supports the idea that protein-induced or cholesterol-associated curvature (and thus packing defects) may induce phase separation in an otherwise disordered fluid membrane. As discussed by Kang *et al*. (2024), we note that the nanodomains induced by packing defects observed in (a leaflet of) the vesicle is not necessarily consistent with lipid rafts, which may be specifically associated with particular sizes, components, ordering, *etc*. For instance, lipid rafts are typically associated with collective (in-membrane) structures greater than 10 nm, while Fig. 4 shows that nanodomains in this study are of the order of 1 nm.

## CONCLUSION

We investigate the effect of increasing concentrations of cholesterol on the functional role of α-Syn, in particular synaptic-like vesicle clustering mediated by α-Syn. Using single-vesicle clustering assay and MD simulation, we find cholesterol modulates vesicle clustering mediated by alpha-Synuclein dependent on concentration in membrane, and this dependence is nonlinear and non-monotonic. In particular cholesterol enhances vesicle clustering with α-Syn for high concentrations, and inhibits vesicle clustering for low cholesterol concentration. Furthermore, MD simulation suggests this enhancement is predominantly driven by enhancement of electrostatic interaction via hydrogen bonds in the protein–membrane system. Hence, our results suggest that α-Syn’s N-terminal region membrane interaction with phospholipid membranes containing variable cholesterol results may increase or decrease vesicle clustering by changing the membrane environment and making membrane binding more or less favorable for α-Syn.

## EXPERIMENTAL METHODS

### Materials

Phospholipids were purchased from Avanti Polar Lipids. Fluorescent dyes were purchased from Life Technologies Corporation. Cholesterol (CHOL) and NeutrAvidin were purchased from Thermo Scientific. Recombinant human α-Syn protein was purchased from Alexotech (Sweden).

#### Lipids

• 1,2-dioleoyl-sn-glycero-3-phosphocholine (DOPC)

• 1,2-dioleoyl-sn-glycero-3-phosphoethanolamine (DOPE)

• 1,2-dioleoyl-sn-glycero-3-phospho-L-serine (DOPS)

• 1,2-dioleoly-sn-glycero-3-phospholethanolamine-N-biotinyl (biotin-PE)

#### Dyes

• DiD (DiIC18(5))

• DiI (DiIC18(3))

#### Other

• Cholesterol

• NeutrAvidin

• α-Syn

### Vesicle preparation

Small unilamellar vesicles (SUVs) have been widely used for *in vitro* studies of protein–lipid system interactions previously (Aryal *et al*. 2021; Bu *et al*. 2018; Cai *et al*. 2019, 2020; Diao *et al*. 2013; Lai *et* *al*. 2023; Liu *et al*. 2021; Tyoe *et al*. 2023; Ulmer *et al*. 2005). We follow the same procedure for SUV preparation of SV-like vesicles as reported previously (Aryal *et al*. 2021; Tyoe *et al*. 2023); for a more detailed description of the experimental procedure see our protocol for quantifying vesicle clustering induced by α-Syn using TIRF (Aryal *et al*. 2021). Table 1 shows the composition of lipid mixtures. Samples are sealed in a vacuum to yield a lipid film. The film was hydrated, then annealed to obtain a unilamellar vesicle solution, which was then extruded through a 50-nm filter to obtain a uniform distribution of SUVs.

### Surface treatment and microfluidic chamber assembly

We follow the same procedure for surface treatment and microfluidic chamber assembly as reported previously (Aryal *et al*. 2021; Tyoe *et al*. 2023). A single sample is comprised of one pre-drilled quartz glass slide and one glass coverslip. These samples are cleaned and the surface of the quartz slide oxidized. The samples are then incubated in the aminosilanization solution below. Samples are then rinsed with water, followed by methanol, and then dried. 100 µL of PEG solution was added to each quartz slide and covered by a treated coverslip. Each sample was then incubated overnight, then rinsed with water and dried, to yield PEGylated slides.

Aminosilanization solution: 100 mL methanol, 5 mL acetic acid, and 1 mL amino saline (3-(2-aminoethylamino)propyl) trimethoxylinane).

PEG solution: 120 mg mPEG, 4 mg biotin-PEG and 700 µL sodium bicarbonate (0.1 mol/L).

Microfluidic channels are constructed by first applying thin strips of double-sided tape on the PEGylated surface of the slide in between the pre-drilled holes. The coverslip was placed on top (treated side down) and sealed on both edges using Epoxy.

### Sample preparation

We follow the same procedure for sample preparation for TIRF imaging as reported previously (Aryal *et al*. 2021; Tyoe *et al*. 2023). After each step, the channel is flushed with HEPES buffer to remove any unbound protein or vesicles.

We added 20 µL of NeutrAvidin solution per microfluidic channel and incubated for 20 min. 100 µL DiD-vesicles was added, followed by incubating for 30 min. 50 µL α-Syn solution was added to each (non-control) channel and incubated for 30 min. 100 µL of DiI-vesicle solution was added and incubated for 30 min.

HEPES buffer: 25 mmol/L HEPES, 100 mmol/L NaCl, pH 7.4.

NeutrAvidin solution: NeutrAvidin 0.1 mg/mL in 10 mmol/L Tris-HCL, 50 mmol/L NaCl, pH 7.5.

### TIRF microscopy and analysis

A representation of our experimental setup for vesicle clustering assay using TIRF microscopy is shown in Fig. 1A. Imaging was done separately for each fluorescent-labelled vesicle, exciting with a green laser (*λ* = 532 nm) for DiI-vesicles and a red laser (*λ* = 633 nm) for DiD-vesicles. Ten images per laser are obtained at random positions per channel. To quantify (DiI-labelled) vesicle clustering, we counted the number of fluorescent sources, using an algorithm described previously (Aryal *et al*. 2021; Tyoe *et al*. 2023). A significance test of protein induced changes in vesicle clustering was performed using the student’s *t*-test.

### Preparation of MD simulation models

**Figure 7:**
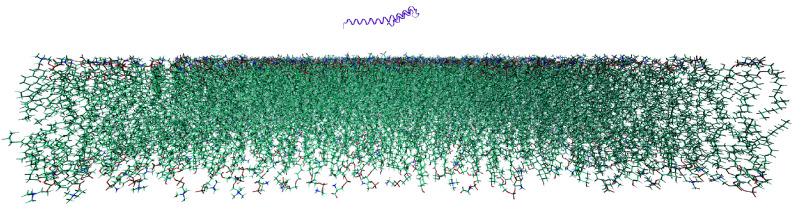
Initial simulation configuration of N-terminal region of α-synuclein (residues 1–60) relative to the center of the lipid bilayer, pre-EM, pre-MD. In the membrane, cyan represents C–C bonds, red represents O-bonds, orange P-bonds, blue N-bonds, and H-bonds are white for a clearer picture of membrane structure. This particular membrane is composed of 12% DOPS, 20% DOPE, and 68% DOPC, corresponding to 0% cholesterol

We follow the same procedure for model preparation as reported previously (Cai *et al*. 2020; Liu *et al*. 2021; Tyoe *et al*. 2023). Structures of simulated lipid bilayers were constructed using CHARMM-GUI (Jo *et al*. 2008; Lee *et al*. 2016). All membrane structures are composed of 2048 lipids. The lipid composition corresponding to 0% CHOL is given by the composition 12% DOPS + 20% DOPE + 68% DOPC; 10% CHOL given by 12% DOPS + 20% DOPE + 58% DOPC + 10% CHOL; and 40% CHOL given by 12% DOPS + 20% DOPE + 28% DOPC + 40% CHOL. In all simulations, a vesicle was approximated as a flat membrane, while the packing density of lipids was fixed to mimic those in a similarly sized curved membrane (of SVs) by applying tension to the flat membrane (Cai *et al*. 2020; Cornell *et al*. 1980; Huang and Mason 1978; Leontiadou *et al*. 2004; Liu *et al*. 2021; Tyoe *et al*. 2023). The system was solvated in a TIP3P water box (Jorgensen *et al*. 1983), to achieve appropriate water density, and typical box sizes are approximately 20 nm × 20 nm × 12 nm. Sodium ions were also added to neutralize the system. The α-Syn structure file was obtained from the RCSB protein data bank, PDB ID: 1XQ8 (Ulmer *et al*. 2005). Only residues 1–60 were used in the simulations, corresponding to the N-terminus of α-Syn. For the initial configuration of the system protein, the protein is positioned parallel to the membrane surface, with an initial separation of approximately 5 nm (Fig. 7).

### MD simulation method

We follow the same procedure for MD simulation as reported previously (Cai *et al*. 2020; Liu *et al*. 2021; Tyoe *et al*. 2023). All-atom MD simulations were performed with GROMACS (Van Der Spoel *et al*. 2005) using the CHARMM36 force field (Klauda *et al*. 2010). All simulations employed periodic boundary conditions, with temperature coupling via the V-rescale algorithm at 310 K (Bussi *et* *al*. 2007). The pressure was coupled to *p_zz_* = 1 bar, *p_xx_* = *p_yy_* = –28 bar for 15 ns via the Parrinello–Rahman method (Parrinello and Rahman 1981), to achieve appropriate lipid packing density, approximately 1.28 lipids/nm^2^. We used the LINCS algorithm to constrain bonds to hydrogen atoms (Hess 2008). The time step used was 2.0 fs. Long-range electrostatic interactions were calculated via the particle-mesh Ewald (PME) summation method (Essmann *et al*. 1995). The distance used for a cutoff of non-bonded interactions was fixed at 12 Å. Energy analyses were performed by averaging interaction energies calculated for an ensemble of protein–lipid systems, in our case four simulations each (with or without cholesterol). In any given ensemble, 0%, 10%, or 40% CHOL, the composition and MD parameters are fixed in all simulations, as well as the initial configuration of the protein and membrane via prescribed restraints in GROMACS. Error bars are given by standard deviations. Structure visualization from Fig. 4A was performed using VMD (Humphrey *et al*. 1996).

### Method for detection of membrane packing defects, calculation of defect size constant

We follow the same procedure for quantification of membrane defects as reported previously (Liu *et al*. 2021; Tyoe *et al*. 2023).

Head group atoms of lipids were projected onto a grid mesh with a grid cell size of 0.1 nm. For each grid cell, our algorithm uses distances from projected atoms and grid cell centers to confirm if a distance inequality is satisfied to define overlap (Liu *et al*. 2021; Tyoe *et al*. 2023). If this inequality is not satisfied, this is defined as no overlap between the grid cell and the projected atom. If there is no overlap between a grid cell and any atoms, the grid cell is identified as a defect with the area of (a grid cell length squared) 0.01 nm^2^. Defect grid cells within a distance of 0.3 nm are identified as the same defect. The area of one defect is thus defined as the total area of all its combined defect grid cells. For more details on the calculation of defect size constant, see Manna *et al*. (Manna and Murarka 2021). Briefly, we vary the size of the defect threshold to create a defect probability distribution as a function of defect size, and fit this distribution with an exponential function $ A{e}^{-\frac{x}{{A}_{0}}} $ for small defects, to obtain a value for A_0_, which is defined to be the defect size constant.

## Conflict of interest

Owen Tyoe, Chinta Aryal and Jiajie Diao declare that they have no conflict of interest.

## References

[bAryal2021] (2021). Lipid species dependent vesicles clustering caused by alpha-Synuclein as revealed by single-vesicle imaging with total internal reflection fluorescence microscopy. Biophys Rep.

[bBu2018] (2018). Cholesterol suppresses membrane leakage by decreasing water penetrability. Soft Matter.

[bBu2017] (2017). N-terminal acetylation preserves α-synuclein from oligomerization by blocking intermolecular hydrogen bonds. ACS Chem Neurosci.

[bBussi2007] (2007). Canonical sampling through velocity rescaling. J Chem Phys.

[bCai2020] (2020). Single-vesicle imaging quantifies calcium's regulation of nanoscale vesicle clustering mediated by α-synuclein. Microsyst Nanoeng.

[bCai2019] (2019). Single-vesicle measurement of protein-induced membrane tethering. Colloids Surf B Biointerfaces.

[bChamberlain2001] (2001). SNARE proteins are highly enriched in lipid rafts in PC12 cells: implications for the spatial control of exocytosis. Proc Natl Acad Sci USA.

[bClayton1998] (1998). The synucleins: a family of proteins involved in synaptic function, plasticity, neurodegeneration and disease. TINS.

[bClayton1999] (1999). Synucleins in synaptic plasticity and neurodegenerative disorders. J Neurosci Res.

[bConway2000] (2000). Fibrils formed *in vitro* from alpha-synuclein and two mutant forms linked to Parkinson's disease are typical amyloid. Biochemistry.

[bCornell1980] (1980). The molecular packing and stability within highly curved phospholipid bilayers. Biochim Biophys Acta.

[bCutler2004] (2004). Involvement of oxidative stress-induced abnormalities in ceramide and cholesterol metabolism in brain aging and Alzheimer's disease. Proc Natl Acad Sci USA.

[bde2013] (2013). Changes in lipid density induce membrane curvature. RSC Adv.

[bDiao2013] (2013). Native alpha-synuclein induces clustering of synaptic-vesicle mimics via binding to phospholipids and synaptobrevin-2/VAMP2. Elife.

[bEliezer2001] (2001). Conformational properties of alpha-synuclein in its free and lipid-associated states. J Mol Biol.

[bEssmann1995] (1995). A smooth particle mesh Ewald method. J Chem Phys.

[bFakhree2016] (2016). The number of α-synuclein proteins per vesicle gives insights into its physiological function. Sci Rep.

[bFantini2013] (2013). The driving force of alpha-synuclein insertion and amyloid channel formation in the plasma membrane of neural cells: key role of ganglioside- and cholesterol-binding domains. Adv Exp Med Biol.

[bFrank2008] (2008). Cholesterol depletion inhibits synaptic transmission and synaptic plasticity in rat hippocampus. Exp Neurol.

[bGil2005] (2005). Synaptic proteins and SNARE complexes are localized in lipid rafts from rat brain synaptosomes. Biochem Biophys Res Commun.

[bGoedert2001] (2001). Alpha-synuclein and neurodegenerative diseases. Nat Rev Neurosci.

[bHess2008] (2008). P-LINCS: a parallel linear constraint solver for molecular simulation. J Chem Theory Comput.

[bHuang1978] (1978). Geometric packing constraints in egg phosphatidylcholine vesicles. Proc Natl Acad Sci USA.

[bHumphrey1996] (1996). VMD: visual molecular dynamics. J Mol Graph.

[bJakubec2021] (2021). Cholesterol-containing lipid nanodiscs promote an α-synuclein binding mode that accelerates oligomerization. FEBS J.

[bJensen2011] (2011). Membrane curvature sensing by amphipathic helices: a single liposome study using α-synuclein and annexin B12. J Biol Chem.

[bJensen1998] (1998). Binding of alpha- synuclein to brain vesicles is abolished by familial Parkinson’s disease mutation. J Biol Chem.

[bJorgensen1983] (1983). Comparison of simple potential functions for simulating liquid water. J Chem Phys.

[bJo2008] (2008). CHARMM-GUI: a web-based graphical user interface for CHARMM. J Comput Chem.

[bKang2024] (2024). Atomistic insight into the lipid nanodomains of synaptic vesicles. J Phys Chem B.

[bKlauda2010] (2010). Update of the CHARMM all-atom additive force field for lipids: validation on six lipid types. J Phys Chem B.

[bKruger1998] (1998). Ala30Pro mutation in the gene encoding alpha- synuclein in Parkinson’s disease. Nat Genetics.

[bLai2023] (2023). Neutral lysophosphatidylcholine mediates α-synuclein-induced synaptic vesicle clustering. Proc Natl Acad Sci USA.

[bLang2007] Lang T (2007) SNARE proteins and 'membrane rafts'. J Physiol 585(Pt 3): 693-698

[bLautenschlager2017] Lautenschlager J, Kaminski CF, Schierle GSK (2017) Alpha-synuclein — regulator of exocytosis, endocytosis, or both? Trends Cell Biol 27: 468–479

[bLee2016] (2016). CHARMM-GUI input generator for NAMD, GROMACS, AMBER, OpenMM, and CHARMM/OpenMM simulations using the CHARMM36 additive force field. J Chem Theory Comput.

[bLeftin2013] (2013). Solid-state ¹³C NMR reveals annealing of raft-like membranes containing cholesterol by the intrinsically disordered protein α-synuclein. J Mol Biol.

[bLeontiadou2004] (2004). Molecular dynamics simulations of hydrophilic pores in lipid bilayers. Biophys J.

[bLiu2021] (2021). Membrane packing defects in synaptic vesicles recruit complexin and synuclein. Phys Chem Chem Phys.

[bLiu2004] (2004). alpha-synuclein produces a long-lasting increase in neurotransmitter release. EMBO J.

[bLokappa2011] (2011). Alpha-synuclein populates both elongated and broken helix states on small unilamellar vesicles. J Biol Chem.

[bMahapatra2021] (2021). Cholesterol in synaptic vesicle membranes regulates the vesicle-binding, function, and aggregation of α-synuclein. J Phys Chem B.

[bManna2021] (2021). Polyunsaturated fatty acid modulates membrane-bound monomeric α-synuclein by modulating membrane microenvironment through preferential interactions. ACS Chem Neurosci.

[bMan2020] (2020). A role of cholesterol in modulating the binding of α-synuclein to synaptic-like vesicles. Front Neurosci.

[bMaroteaux1988] (1988). Synuclein — a neuron-specific protein localized to the nucleus and presynaptic nerve-terminal. J Neurosci.

[bMcClain2023] (2023). Biologically representative lipid-coated gold nanoparticles and phospholipid vesicles for the study of alpha-synuclein/membrane interactions. ACS Nano.

[bMiddleton2010] (2010). Effects of curvature and composition on alpha-synuclein binding to lipid vesicles. Biophys J.

[bNecula2003] (2003). Rapid anionic micelle-mediated alpha-synuclein fibrillization *in vitro*. J Biol Chem.

[bNuscher2004] (2004). Alpha-synuclein has a high affinity for packing defects in a bilayer membrane: a thermodynamics study. J Biol Chem.

[bParrinello1981] (1981). Polymorphic transitions in single-crystals — a new molecular-dynamics method. J Appl Phys.

[bPerlmutter2009] (2009). Curvature dynamics of alpha-synuclein familial Parkinson disease mutants: molecular simulations of the micelle- and bilayer-bound forms. J Biol Chem.

[bQi2023] (2023). Influence of cholesterol on the membrane binding and conformation of α-synuclein. J Phys Chem B.

[bSerpell2000] (2000). Fiber diffraction of synthetic alpha- synuclein filaments shows amyloid- like cross- beta conformation. Proc Natl Acad Sci USA.

[bSpillantini2000] (2000). The alpha-synucleinopathies: Parkinson's disease, dementia with Lewy bodies, and multiple system atrophy. Ann N Y Acad Sci.

[bStefanovic2015] (2015). Alpha-synuclein amyloid oligomers act as multivalent nanoparticles to cause hemifusion in negatively charged vesicles. Small.

[bTang2024] (2024). Folding of N-terminally acetylated α-synuclein upon interaction with lipid membranes. Biophys J.

[bTsigelny2015] (2015). Molecular determinants of α-synuclein mutants' oligomerization and membrane interactions. ACS Chem Neurosci.

[bTyoe2023] (2023). Docosahexaenoic acid promotes vesicle clustering mediated by alpha-synuclein via electrostatic interaction. Eur Phys J E Soft Matter.

[bUlmer2005] (2005). Structure and dynamics of micelle-bound human alpha-synuclein. J Biol Chem.

[bValenza2007] (2007). Cholesterol biosynthesis pathway is disturbed in YAC128 mice and is modulated by huntingtin mutation. Hum Mol Genet.

[bVamparys2013] (2013). Conical lipids in flat bilayers induce packing defects similar to that induced by positive curvature. Biophys J.

[bvan2004] (2004). Nonbilayer lipids affect peripheral and integral membrane proteins via changes in the lateral pressure profile. Biochim Biophys Acta.

[bVan2005] (2005). GROMACS: fast, flexible, and free. J Comput Chem.

[bvan2015] van Maarschalkerweerd A, Vetri V, Vestergaard B (2015) Cholesterol facilitates interactions between α-synuclein oligomers and charge-neutral membranes. FEBS Lett 589(19 Pt B): 2661−2667

[bVermaas2014] (2014). Conformational heterogeneity of α-synuclein in membrane. Biochim Biophys Acta.

[bWang2016] (2016). Versatile structures of alpha-synuclein. Front Mol Neurosci.

[bWu2020] (2020). O-GlcNAcylation inhibits the oligomerization of alpha-synuclein by declining intermolecular hydrogen bonds through a steric effect. Phys Biol.

[bZhu2003] (2003). The association of alpha- synuclein with membranes affects bilayer structure, stability, and fibril formation. J Biol Chem.

